# The urban–rural differential in the association between household wealth index and anemia among women in reproductive age in Ethiopia, 2016

**DOI:** 10.1186/s12905-021-01461-8

**Published:** 2021-08-25

**Authors:** Teshager Weldegiorgis Abate, Biruk Getahun, Mekuriaw Mesfin Birhan, Getasew Mulatu Aknaw, Sefealem Assefa Belay, Dessalegn Demeke, Dagninet Derebe Abie, Adela Memberu Alemu, Yirga Mengiste

**Affiliations:** 1 Department of Adult Health Nursing, School of Health Science, College of Medicine and Health Science, Bahir Dar University, P.O. Box 79, Bahir Dar, Ethiopia; 2 Department of Human Physiology, School of Medicine, College of Medicine and Health Science, Bahir Dar University, P.O. Box 79, Bahir Dar, Ethiopia; 3 Department of Emergency and Critical Care Nursing, School of Health Science, College of Medicine and Health Science, Bahir Dar University, P.O. Box 79, Bahir Dar, Ethiopia; 4 Department of Human Biochemistry, School of Medicine, College of Medicine and Health Science, Bahir Dar University, P.O. Box 79, Bahir Dar, Ethiopia; 5 Department of Pharmacy, School of Health Science, College of Medicine and Health Science, Bahir Dar University, P.O. Box 79, Bahir Dar, Ethiopia; 6 Department of Gynecology, School of Medicine, College of Medicine and Health Science, Bahir Dar University, P.O. Box 79, Bahir Dar, Ethiopia

**Keywords:** Anemia, Women, Reproductive age, Factors, Urban, Rural

## Abstract

**Background:**

Anemia is more prevalent among women, and it is a moderate public health problem in Ethiopia. The wealth status and place of residence of a woman have implications on the intervention of anemia. Studies that examined the relationship between women’s wealth index status and residency in Ethiopia are scarce. We aimed to identify the urban–rural differential in the association between household wealth index and anemia among women of childbearing age in Ethiopia.

**Method:**

A cross-sectional design was employed with a nationally representative sample of 14,100 women aged 15–49-year-old from the Ethiopian demographic and health survey conducted in 2016. We used the two-stage sampling method to select the sample size. The primary outcome was anemia in women of childbearing age. A hemoglobin level of below 11 g/dl for pregnant women and 12 g/dl for non-pregnant women was the indicator of anemia. Using a three-level random intercept model to explore associated factors at the individual and household levels quantified the observed and unobserved variations between household wealth index and residence on anemia.

**Results:**

Women belonging to a lower household wealth index category were more anemic (29.6%) than those middle and above wealth index categories. Women who lived in rural areas (25.5%) were prone to anemia than those who lived in urban areas (17.5%). The odds of anemia were significantly higher in women of the low household wealth category who living in rural compared to women of the middle and above household wealth category who living in urban (AOR = 1.37, 95% CI 1.14–1.65, *P* < 0.001).

**Conclusion:**

In this study, anemia is more common among women who live in rural with the low house wealth category**.** Therefore, novel public health interventions should target women who live in rural areas with the lowest household wealth status.

## Introduction

Anemia is a health condition characterized by a low level of hemoglobin (HGB) in which blood has fewer red blood cells (RBC). A low HGB level impairs blood from delivering oxygen to the body tissues [1]. The causes of anemia are a genetic defect, infections (malaria, hookworm, and bone marrow disease), deficiency of iron, vitamins, folate, copper, and total nutritional deficiencies. Nutritional deficiency anemia is the most common type [2, 3].

Anemia is associated with a defect in birth outcomes including miscarriage, preterm delivery, placental abruption, a low birth weight, higher risk of prenatal and maternal mortality [6, 7]. Anemia can reduce physical activities, cognitive capacities, and reduced work productivity [8]. Anemia, the most public health concern worldwide, affected 27% (1.93 billion people) of the world’s population in 2013. Hence, it is a common public health issue and accounts for more than 89% of the burden in the developing countries. According to 2011 the WHO estimated, anemia affects around 800 million children and women of reproductive age globally [9, 10].

The global prevalence of anemia in pregnant women was 38.2% and for all women of reproductive age was 29.4% [5, 9]. In Africa, anemia affects 35% of women of reproductive age [9, 11]. Its prevalence is even higher in low-income countries such as Ethiopia due to several contributing factors [12, 13]. The symptoms of anemia are strongly associated with the quality of life, a poor-quality diet due to poverty, socioeconomic status, residence, education, and pregnancy status [4]. Besides, due to sex-specific experiences, such as pregnancy, bleeding during childbirth, lactation, and menstruation in women of reproductive age (15–49 years), there is an increased risk of developing anemia as compared to their male counterparts [14].

Anemia is diagnosed when hemoglobin levels fall below 12 g/dl in adult non-pregnant women and below 11 g/dl during pregnancy [1]. The two main causes of anemia are classified by timing; namely immediate causes and distal causes. The proximal factors are mainly attributable to micronutrient deficiencies [15], physiological adaptations during pregnancy [16], and infections such as malaria [17], hookworm, and HIV [18]. Besides these immediate risk factors of anemia, distal factors that operate at the household and community levels include maternal age [4], education status [13], marital status, occupation [19], rural/urban residence [20], household wealth index [18, 21], hormonal contraceptive use, and body mass index [10, 22, 23].

Former studies in Ethiopia focused on children, pregnant and lactating women’s nutrition, and associated factors [12, 24, 25] and identified factors linked to anemia such as residence, wealth status, and modern contraceptive users. However, these studies are small-scale and are limited to specific localities [12, 21, 25–28]. Many former studies showed us the wealth index and residence area are significantly associated with anemia separately [29]. These former studies did not explain the urban–rural differentiation on the interaction between anemia and wealth index.

Therefore, this study aims to analyze the urban–rural differential in the association between household wealth index and anemia among women of reproductive age in Ethiopia. The Ethiopian Demographic and Health Survey (EDHS) is the only source of compressive national data on several characteristics of women of reproductive age (15–49 years). The authors decided to investigate the differentials in anemia among women of reproductive age, using data from the 2016 EDHS.

## Method and materials

### Data source, study design, sample size, and sampling procedures

This study used data from the 2016 Ethiopian Demographic and Health Survey (EDHS). The EDHS was designed to provide representative data on various health indicators for the whole country across the two city administrations and nine regional states. This survey was conducted by the United States Agency for International Development (USAID) in collaboration with the Ministry of Health of Ethiopia and other partner organizations [30].

**Fig. 1:**
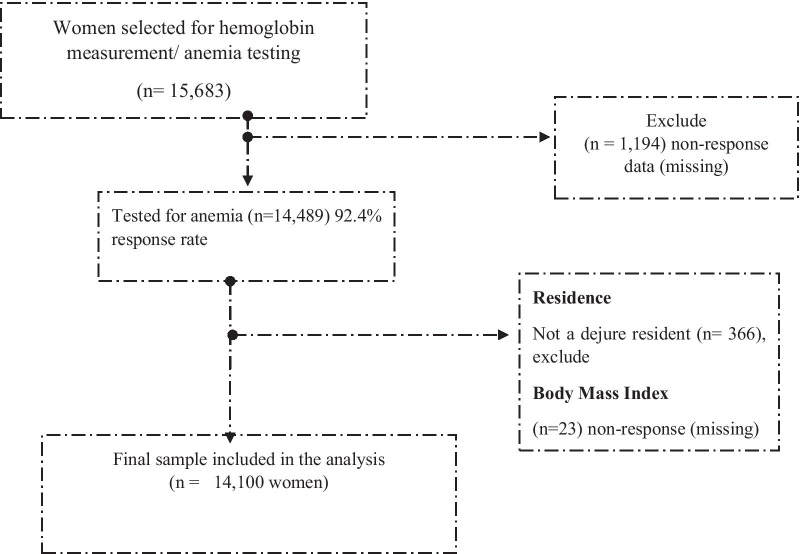
Schematic presentation of selecting a sample from 2016 EDHS data

The 2016 EDHS sample design involved a probabilistic two-stage sampling. Each region was stratified into urban and rural areas, yielding 21 sampling strata. Samples of Enumeration Areas (EAs) were selected independently in each stratum in two stages. Implicit stratification and proportional allocation use at each of the lower administrative levels by sorting the sampling. Women aged 15–49 years who were in the selected households at the time of the survey were eligible for participation. A total of 15,683 women aged 15–49 years were selected for anemia testing in the 2016 EDHS. Almost all women, 92.4% (14,489), were tested for anemia. The final sample size for this study was 14,100 women (Fig. 1).

### Variable descriptions

Anemia is an outcome variable for this study. Following WHO recommendations [1, 31] anemia in non-pregnant women was defined as any anemia if their hemoglobin concentration was below 12·0 g/dL, moderate below 11·0 g/dL, and severe if hemoglobin was below 8·0 g/dL. Anemia in pregnant women was defined by hemoglobin levels below 11.0 g/dL and anemia, non-pregnant women was defined by hemoglobin was below 12.0 g/dL [30, 32]. The main anemia associated variable of interest were the household wealth index and place of residence (Table 1) [4, 10, 18, 20, 22, 23, 33].

**Table 1:** Description of explanatory variables used in the analysis of anemia among reproductive women in Ethiopia 2016 (n = 14,100)

Variables	Overall number (wt%)	Variables	Overall number (wt%)
Mother's age		Employment status	
15–24	5631 (38.4)	Yes	9078 (66.8)
25–34	4622 (34.1)	No	5022 (33.2)
35–49	3847 (27.4)	House hold wealth index	
Mother's marital status		Poor	5487 (35.3)
Unmarried	3662 (24.9)	Middle	1861 (19.5)
Married	9024 (66.0)	Rich	6752 (45.2)
Others*	1414 (9.1)	Body mass index	
Mother's education		Under weight	3399 (21.2)
None	6467 (48.7)	Normal weight	9084 (71.1)
Primary	4740 (35.2)	Overweight	1617 (7.7)
Secondary or above	2893 (16.1)	Use hormonal contraceptives	
Place of residence		No	11,407 (76.8)
Urban	4563 (21.2)	Yes	2693 (23.2)
Rural	9537 (78.8)		

wt%, weighted percent

^*^Divorced, widowed and no longer living together, and Overweight and obese was grouped due to small number of cases

### Data analysis

All the statistical analyses were done while taking into account the complex design of the survey. All the estimated sample was considered after weighing by the sample weighing factor and taking the cluster design of the study. While it ensures data representativeness at both national and sub-national levels, the DHS sampling procedure was over-represented small regions. Thus, sample weighting was applied to compensate for the unequal probability of sample selection and ensure the sample resembles the population distribution. The final weighted sample size is 14,100 women [30]. Data were analyzed by Chi-square and binary logistic regression models using SPSS software for Windows Version 20. We described the characteristics of the study population and cross-tabulated our dependent variables with the explanatory variables.

The Chi-square were carried out to test the association between the dependent and independent variables. After identifying the association between anemia and independent variables in Chi-square. Only one independent variable include at a time in the regression equation to examine their relationship with anemia. Both the wealth index and place of residence were introduced into the regression equation simultaneously to assess their association with anemia (*P*-value < 0.2). A significant interaction effect would indicate the residence factor on the relationship between the household wealth index and the presence of anemia.

Finally, multivariate logistic regression analyses performed to identify the independent associations of explanatory variables with the outcomes of interest, providing Adjusted Odds ratios (AOR) and 95% confidence intervals (CI). Pearson, Hosmer, and Lemeshow (HL) test to check for model fit (Hosmer & Lemeshow), and the final model had a better HL chi-square value and *P*-value. The level of statistical significance sets at 5%.

### Ethical consideration

The EDSH survey was carried out by the central statistical agency of Ethiopia. The study protocol and data collection instruments were reviewed for adherence to ethical standards by the Ethiopian Health and Nutrition Research Institute. All study participants were required to provide informed written consent prior to undertaking the survey. Privacy and confidentiality were ensured following the ethical requirements of research. The study involved minimal risk for study participants [30]. Since this study was a secondary analysis of the Ethiopian Demographic and Health surveys (EDHS) data, which are publicly available, our study did not require any ethics approval. DHS program authorization was requested to download the dataset.

## Result

### Socio-demographic characteristics of the sample

In this study, a total of 14,100 women were included from nine regional states and two city administrations in Ethiopia. The mean age was 28.2 ± 0.2 and majority of anemic women lived in rural areas (25.5%). More women of low household wealth index categories were anemic (29.6%) compared to those classified rich wealth index categories. More women who live in rural areas were (25.5%) were anemic than compared to those who live in urban areas (Table 2).

**Table 2:** Characteristics of the study sample and the status of anemia in women of reproductive age (n = 14,100)

Variables	Anemia		*P*-value
	Yes n (wt%)	No n (wt%)	
Mother's age			0.02
15–24	1432 (21.9)	4199 (78.1)	
25–34	1361 (25.6)	3261 (74.4)	
35–49	1054 (24.2)	2793 (75.8)	
Mother's marital status			< 0.001*
Unmarried	746 (17.9)	2916 (82.1)	
Married	2759 (26.4)	6265 (73.6)	
Others**	342 (20.8)	1072 (79.2)	
Mother's education			< 0.001*
None	2207 (27.9)	4260 (72.1)	
Primary	1111 (21.5)	3629(78.5)	
Secondary or above	529 (16.2)	2364 (83.8)	
Place of residence			< 0.001*
Urban	905 (17.1)	3658 (82.9)	
Rural	2942 (25.5)	6595 (74.5)	
Employment status			0.003*
Yes	1139 (21.4)	3883 (78.6)	
No	2708 (24.9)	6370 (75.1)	
House hold wealth index			< 0.001*
Poor	2027 (29.6)	3460 (70.6)	
Middle	477 (23.8)	1384 (76.2)	
Rich	1343 (19.2)	5409 (80.8)	
Body mass index			0.035
Under weight	1096 (26.2)	2303 (73.8)	
Normal weight	2404 (23.3)	6680 (76.7)	
Overweight	347 (21.2)	1270 (78.8)	
Use hormonal contraceptive			< 0.001*
No	3358 (25.3)	8049 (7.7)	
Yes	489 (18.8)	2204 (81.2)	

wt.%, weighted percent

^*^P-value less than 0.005

^**^Divorced, widowed and no longer living together

### Association wealth index and anemia stratified by place of residency

Variables included in the bivariate regression analysis based on their association in the chi-squared analyzes (Table 3). The bivariate model provides the unadjusted odds ratio of the association between anemia and wealth index with the different explanatory variables. Seven variables were significantly associated with anemia in this model. Six variables remained statistically significant in the final multiple regression model controlling all explanatory variables.

**Table 3:** Association wealth index and anemia stratified by place of residence (n = 14,100)

Variables	Rural		Urban	
	AOR (95% CI)	*P*-value	AOR (95% CI)	*P*-value
Mother's age				
15–24	1.13 (1.057–1.68)	0.017	0.81 (0.59–1.12)	0.199
24–34	1.27 (1.05–1.52)	0.013	0.75 (0.56–1.01)	0.58
35–49*	1	1		1
Mother's education				
None	1.59 (1.13–2.23)	0.008	0.98 (0.68–1.41)	0.906
Primary	1.27 (0.94–1.71)	0.122	1.14 (0.75–1.730)	0.539
Secondary or above*		1		1
Marital status				
Unmarried	0.84 (90.62–1.14)	0.268	0.81 (0.50–1.2)	0.284
Married	1.53 (1.20–1.97)	0.001	0.98 (0.71–1.35)	0.901
Others**	1	1		1
Wealth index				
Poor	1.37 (1.14–1.65)	0.001	0.86 (0.46–1.62)	0.648
Middle	1.11 (0.93–1.33)	0.247	1.10 (0.43–2.77)	0.843
Rich*	1	1		1
Use hormonal contraceptive				
Yes*	1	1		1
No	1.67 (1.41–1.95)	< 0.001	1.53 (1.13–2.07)	0.006
Body mass index				
Under weight	1.06 (0.74–1.52)	0.752	1.10 (0.71–1.7)	0.674
Normal weight	0.89 (0.65–1.24)	0.507	1.10 (0.72–1.58)	0.741
Overweight*	1			
Employment status				
Yes*	1			
No	1.04 (0.89–1.21)	0.634	1.159 (0.87–1.51)	0.326

wt%, weighted percent; OR, odds ratio; CI, confidence interval

^*^Reference group

^**^Divorced, widowed and no longer living together

More women of low household wealth index and live in a rural area were anemic compared to women of high house wealth index and living in urban. For instance, women who were a low household wealth index and rural residence category were 1.37 times more likely to anemia than their counterparts who belong to the wealthy class and urban residency. Also, in the interactive expression, being a low household health index played a role in the relationship between residency and wealth index and level of anemia. In the interactive model, the odds ratio of anemia was higher for women who lived in rural rather than urban residences (Table 3).

Other predictors of anemia were women who were not using hormonal contraceptives, younger age category, having no education, and married women. The data also show using the wealth index and women’s residency jointly or interactively in the regression equation generated the same predictors with differences in their pattern of the odds ratio. The likelihood of anemia was significantly higher among the lower wealth class women living in a rural area in Ethiopia (Table 3).

## Discussion

This study uses a country-level, representative data in Ethiopia to describe urban–rural differential in the association between household wealth index and anemia among women in the reproductive age group. This study provides evidence linking specific anemia factors related to the reproductive age group of women. Since successful anemia prevention strategies should rely on evidence-based approaches, the result of this study should represent recommended public health interventions and policies aimed at a target age group. And then reduce the burden and consequences of anemia in this group.

The prevalence of anemia among women in Ethiopia is 24% in 2016 [30], making it a moderate public health problem according to the WHO threshold [34]. The results suggest that level of household wealth was associated with anemia in women who live in rural areas. The prevalence of anemia in women varies significantly between urban and rural and region to region in the communities [18]. In Ethiopia, anemia among the reproductive age group increased 17% in 2011 to 24% in 2016 [30].

In our study, the authors showed that the impact of economic status and residency was the single contributor to anemia among reproductive-age women in Ethiopia. This study found that 29.6% of women of reproductive age who come from poor house wealth status were anemic. Of the total participants, 25.1% of the reproductive age group of women who live in rural areas experience anemia. The prevalence of anemia (29.6% and 25.1%) revealed by this study is higher than the national average (24%). The magnitude of anemia in rural residency is higher than in urban. In this study, the wealth status of women’s households associated with the development of anemia. Women who had the lowest wealth statuses (AOR = 1.37; 95% CI 1.14–1.65) increase the chance of developing anemia in rural residency. This finding is consistent with other studies in low and middle-income countries. These studies found that the associated factors of anemia among women living in a poor wealth status have anemia than those living in the heights wealth status [11, 18, 20, 22, 23, 33].

Because those from the lowest wealth status in the countryside cannot purchase the quality or quantity of foods when compared with those from higher wealth status. Lower-income households purchase less healthful foods when compared with higher wealth. It is plausible that women from low wealth status homes, as with urban residents, may have diversified diets and supplements, thereby decreasing their chance of nutrition-related anemia [32, 35, 36].

Another fact also the most reported dietary consumption of the household in rural residency is monotonous foodstuff and low diet diversification [1, 32]. Women who belong to a low wealth status experienced inadequate food resources, food did not last, did not afford balanced meals, reduced meal size, or skipped meals [37, 38]. The women’s knowledge of different vitamin and mineral-containing foods and their benefit may be other contributors [19].

We observed a place of residency-specific variations in the association of anemia among women with similar household wealth status. Residency may mediate anemia difference in the same wealth status in the Ethiopian reproductive women age group. Our study showed associations between the low socioeconomic status of those who live in rural and anemia. In rural residency, women have attributed disparities in health service provision and access, disease risk, fertility preferences [37, 39, 40]. On the other round, farming can increase the chance of anemia-inducing infectious diseases such as malaria, intestinal parasites like schistosomiasis, and hookworm, and such exposures may be more common in rural residency [33, 41].

In this study, there were significant differences in anemia between urban and rural areas with similar socioeconomic status. The cause of urban–rural difference was related to the sociocultural conditions of the participant. The potential cause of this may be restricted access to diverse micronutrient-rich diets (food taboo), not access to mass media, and nutrition information for women who live in rural can exacerbate anemia. Multiple deficiencies tend to cluster within individuals, and the synergistic effect of these deficiencies plays in the development of anemia in rural residency [39, 42, 45, 46]. Other than residency and socioeconomic status, potential predictors of anemia include being within the younger age category, having no education, being married, and not using hormonal contraceptives, which have been reported in several other studies [4, 9, 10, 18, 23, 24, 33, 46].

### Strengths and limitations

This study used population-based data with large sample size and representative of all regions of Ethiopia. Due to the cross-sectional nature of the EDHS data, the cause-effect and the temporal relationship could not be established based on these study findings. Similarly, essential factors such as family size, gravidity, and parity, religion, current breastfeeding, smoking, and HIV factors were not incorporated these variables in the analysis.

## Conclusion

This study should represent recommended public health interventions and policies aiming at a target age group (rural residency) and reduce the burden and consequences of anemia in this age group in the nation. This study provides evidence linking specific anemia factors related to women's reproductive age group who live in the countryside. Since the driver of anemia in this study is a place of residency affecting women of reproductive age groups, novel public health interventions should consider women who live in rural with the lowest household wealth status.

## Data Availability

All data generated or analyzed during this study are included in this the manuscript.
